# How Do You Know If You Were Mind Wandering? Dissociating Explicit Memories of Off Task Thought From Subjective Feelings of Inattention

**DOI:** 10.1162/opmi_a_00142

**Published:** 2024-05-10

**Authors:** Nathan K. Mathews, Umer Bin Faiz, Nicholaus P. Brosowsky

**Affiliations:** Department of Psychology, University of Manitoba, Winnipeg, MB, Canada

**Keywords:** mind wandering, task-unrelated thought, inattention, metacognition

## Abstract

Mind wandering is a common experience in which your attention drifts away from the task at hand and toward task-unrelated thoughts. To measure mind wandering we typically use experience sampling and retrospective self-reports, which require participants to make metacognitive judgments about their immediately preceding attentional states. In the current study, we aimed to better understand how people come to make such judgments by introducing a novel distinction between explicit memories of off task thought and subjective feelings of inattention. Across two preregistered experiments, we found that participants often indicated they were “off task” and yet had no memory of the content of their thoughts—though, they were less common than remembered experiences. Critically, remembered experiences of mind wandering and subjective feelings of inattention differed in their behavioral correlates. In Experiment 1, we found that only the frequency of remembered mind wandering varied with task demands. In contrast, only subjective feelings of inattention were associated with poor performance (Experiments 1 and 2) and individual differences in executive functioning (Experiment 2). These results suggest that the phenomenology of mind wandering may differ depending on how the experiences are brought about (e.g., executive functioning errors versus excess attentional resources), and provide preliminary evidence of the importance of measuring subjective feelings of inattention when assessing mind wandering.

## INTRODUCTION

Mind wandering is a ubiquitous phenomenon where individuals spontaneously shift their attention from the task at hand to unrelated thoughts (Smallwood & Schooler, 2006). For instance, while driving home from work, you may find yourself lost in thought about the upcoming weekend and arrive home without any memory of your journey. Recent research has largely focused on the consequences of mind wandering, with the majority of studies demonstrating its negative effects (for a review, see Mooneyham & Schooler, 2013), and some recent efforts attempting to identify positive correlates (e.g., Baird et al., 2012; Gable et al., 2019; Murray et al., 2021; Smith et al., 2022). To measure mind wandering, researchers rely on experience sampling and self-report methods that require participants to make metacognitive judgments of their attentional states, yet little consideration has been given to understanding *how* individuals actively form these judgments (e.g., Reisberg & McLean, 1985). In the current study, we aimed to address this gap by investigating the metacognitive mechanisms that enable judgments about mind wandering. Specifically, we hypothesized that people rely on two types of information to make such judgments: explicit memories of off task thought and subjective feelings of inattention. Using this conceptual framework, we conducted two experiments to explore the connections between these inattention experiences, behavioural correlates of inattention, and theories of mind wandering.

Over the past two decades, there has been a renewed interest in the concept of mind wandering, with a significant debate arising regarding its underlying causes (Callard et al., 2013). Attentional resource accounts posit that mind wandering is contingent on the availability of executive resources (Smallwood & Schooler, 2006). As per this perspective, the ability to maintain, update, and manipulate information in working memory depends on a limited pool of resources that is depleted and replenished over time (e.g., Popov & Reder, 2020). Furthermore, since both task engagement and mind wandering compete for the same resources, the extent to which individuals can engage in mind wandering is determined by the availability of those resources. This perspective gains support from the commonly observed impact of task demands on mind wandering. Specifically, reducing the demands of a task—whether through increased predictability (Seli et al., 2016), lower working memory load (Brosowsky et al., 2021b; Konishi et al., 2015; Robison et al., 2020; Rummel & Boywitt, 2014; Seli et al., 2018), reduced perceptual load (Forster & Lavie, 2009), or decreased task complexity (Randall et al., 2014)—tends to lead to an increase in the frequency of mind wandering. Also consistent with this claim, mind wandering impairs explicit learning, thought to require attentional resources, but not implicit learning, which does not attentional resources (Brosowsky et al., 2021a).

In contrast, the Executive Control Failure account, as proposed by McVay and Kane (2010), posits that mind wandering arises due to the failure of executive control to suppress salient thoughts about ongoing concerns during focal task engagement. This viewpoint is corroborated by evidence that mind wandering is negatively associated with performance on sustained attention tasks (e.g., Brosowsky et al., 2023) and measures of executive functioning, such as working memory capacity (McVay & Kane, 2009, 2012).

Although the attentional resources and executive control failure accounts are typically presented as competing viewpoints (e.g., McVay & Kane, 2010), some have suggested that they could be compatible and even complementary (e.g., Smallwood, 2013). For instance, according to Thomson et al.’s (2015) Resource-Control Account, it is the executive control processes that are responsible for allocating attentional resources across tasks. Under this view, mind wandering can occur when there is an excess of available resources, which is automatically allocated to mind wandering without interfering with primary task performance. However, when a disproportionate number of resources are allocated to mind wandering it impairs task performance—what we would call an executive control failure. Thus, mind wandering can be explained by *either* an inappropriate allocation of task-required resources due to executive control failure or the allocation of excess resources.

Due to its covert nature (e.g., Nisbett & Wilson, 1977), however, it can be challenging to experimentally distinguish between different theoretical accounts of mind wandering. To gain insights into mind wandering, researchers typically use experience sampling combined with retrospective self-reports (Weinstein, 2018). In the lab, for instance, participants complete sustained attention tasks that are interrupted at unpredictable intervals to answer questions about their thoughts just prior to the interruption (the probe-caught method; Smallwood & Schooler, 2006)^1^. There are a variety of ways researchers can ask about mind wandering (e.g., Weinstein, 2018), however, most often, participants are asked to categorize their immediately preceding thoughts as “on task” or “off task”. This straightforward approach has been instrumental in advancing our understanding of mind wandering, but assessing the validity and reliability measures of self-reported inattention has proven challenging (for a review, see Kane et al., 2021).

Recently, researchers have begun to address the difficulties in creating accurate and reliable thought probes (Kane et al., 2021). However, less consideration has been given to the metacognitive obstacles that participants may encounter when reporting on their thoughts (e.g., Koriat, 2007; Nisbett & Wilson, 1977). For instance, Schooler (2002) notes that turning metacognition toward a prior mind wandering experience can be quite challenging because people often lack meta-awareness during such episodes (see also, Schooler et al., 2011). They also highlight the possibility of self-reports misrepresenting contents of consciousness due to ambiguity in the initial mental experience or translation errors (i.e., errors due to verbally describing a non-verbal experience). In addition, memory limitations may also influence how past mental experiences are evaluated. Short-term memory capacity and duration limits are well-known, and numerous experiments have shown that complete forgetting can happen in as little as one or two seconds (i.e., rapid forgetting; see Lewandowsky et al., 2009). As a result, reporting your thoughts and mental experiences from even a few seconds ago may be more difficult than commonly assumed.

Despite the aforementioned metacognitive challenges, there is a notable lack of knowledge regarding how individuals form judgments about their inattention (Reisberg & McLean, 1985), especially in the context of mind wandering (Schooler, 2002). To bridge this gap, we developed a novel framework to investigate the metacognition of mind wandering. We hypothesized that people rely on two types of information to form judgments about their mind wandering. First, we hypothesized that people use explicit memories of their prior thoughts to determine if they were mind wandering; this assumption is implicit in most categorical thought probes (e.g., Kane et al., 2021). Second, we hypothesized that when individuals lack explicit memory of their thoughts, they may rely on subjective feelings of inattention. A subjective feeling of inattention, here, is defined simply as a state in which individuals have a subjective experience that they were inattentive or mind wandering despite having no explicit recollection of the content of their thoughts. Although the notion that individuals use heuristics to make judgments about their mental experiences has a long history in cognitive psychology, feelings of inattention have not been previously explored in the context of mind wandering. The theoretical foundation of this framework is rooted in theories of metacognitive judgments of memory (Whittlesea et al., 1990; Whittlesea & Williams, 2001a) and learning (Koriat, 2007).

The idea of determining whether one’s attention was off-task based on a ‘feeling of inattention’—as opposed to an explicit memory—partially intersects with the concept of ‘mind blanking’. Mind blanking occurs when participants report that their ‘mind was blank’ or they experienced a ‘gap in consciousness’ just before a thought probe (e.g., Ward & Wegner, 2013). Indeed, if someone genuinely had ‘no thoughts’ at that time, they would naturally report an absence of memory regarding their thoughts. However, participants might report having no memory, not because they believe they were unconscious or had ‘no thoughts’, but rather because they have forgotten what they were thinking about. Similarly, while experiencing a ‘blank mind’ might lead to feelings of inattention, it is unlikely to be the sole cause of such feelings. For example, performance disfluency or a discrepancy between actual performance and normative expectations about performance may also induce feelings of inattention (Brosowsky et al., 2021a; see also, Whittlesea & Williams, 2000, 2001a). Thus, while conceptually distinct, ‘feelings of inattention’ are likely to capture mind blanking in addition to a wider range of inattention experiences.

To explore our framework, we created a thought probe which distinguishes between explicit memories of off task thought and subjective feelings of inattention. Over the course of two preregistered experiments, we investigated whether such a distinction is meaningful in terms of describing the phenomenology of mind wandering and elucidating the metacognitive processes that enable self-reports of mind wandering. Additionally, we examined behavioral correlates of mind wandering to better understand how subjective feelings of inattention and remembered experiences correspond with current theories of mind wandering.

## EXPERIMENT 1

In Experiment 1, we investigated subjective feelings of inattention and remembered experiences of mind wandering in a well-studied experimental context, using a sustained attention to response task (Robertson et al., 1997) with a demand manipulation (Seli et al., 2016). On every trial, participants were briefly presented a number. Participants responded to every number with a keypress, except the number three, which they were to withhold their response for. Half of the participants received a ‘high demand’ version of the task, where the sequence of numbers was unpredictable. The other half received a ‘low demand’ version of the task, where the numbers appeared sequentially, in a predictable manner (Seli et al., 2016). At irregular intervals, the task paused, and participants were asked to indicate whether their thoughts, just prior to the thought probe, were (1) “On task”, (2) “Off task and I DO remember what I was thinking about” or (3) “Off task, but I DO NOT remember what I was thinking about”. As in Seli et al. (2016), we expected that overall mind wandering would be higher in the low versus high demand groups, and performance would be negatively associated with mind wandering rates.

### Methods

#### Sample Size Justification.

Seli et al. (2016) observed an effect size of partial eta squared of .2 (Cohen’s *f* = .5). Using G*Power, we estimated that with 75 participants per group we would detect interaction effects as small as .15 with 80% power and medium-sized interaction effects (*f* = .25) with 99.76% power (https://osf.io/acrz9)^2^.

#### Participants.

153 University of Manitoba undergraduate students participated in the experiment for course credit through the university SONA subject pool. Participants were all students ages 18 to 45 (mean = 20, *SD* = 3.3; median = 19). When asked about their gender using a free response, 102 participants identified as a woman or female, 48 as a man or male, 2 as non-binary (including “non-binary” and “gender fluid” responses), and 1 participant did not provide a response. When asked about handedness, 119 participants indicated they were right-hand dominant, 19 were left-hand dominant, 5 participants indicated “both”, and 10 participants did not provide a response.

#### Task Materials.

All tasks were programmed in JavaScript using jsPsych (de Leeuw, 2015). All materials can be found online at https://osf.io/8rfbt/.

##### Sustained Attention to Response Task (Robertson et al., 1997; Seli et al., 2016).

For the SART, participants were presented a number on the screen (1–9) and were required to press the space bar for every number except the number 3. Numbers were presented for 350 ms followed by a mask presented for 950 ms (an “X” within a circle). Participants could respond at any time during the trial. The font-size of the numbers were randomly selected on each trial and could have been presented at 36 pt, 48 pt, 60 pt, 72 pt, 84 pt, 96 pt, 108 pt, 120 pt, 132 pt font-sizes. The mask was always presented in 120 pt font. In the ‘high’ demand version of the task, the numbers were presented in a random order. In the ‘low’ demand version, the numbers were presented sequentially, starting at random point in the sequence as in Seli et al. (2016).

First, participants completed a practice session of 45 SART trials and one thought probe. If an error was made during the practice session error feedback was displayed with a reminder to respond to every number except the number 3 as quickly and as accurately as possible. At the end of the practice block, a summary of their performance was provided including average response time, average accuracy on Go trials and average accuracy on No-Go trials.

The experimental session consisted of 24 blocks, each consisting of 45 SART trials and a thought probe. No performance feedback was provided during the experimental session, however, if participants failed to respond to five successive go trials, the experiment paused, and participants were informed that the experiment was paused because they failed to respond too many times. Participants were required to press the space bar to continue.

The thought probe was always randomly presented between trials 9 and 36 of each block. On each thought probe, participants were asked to indicate which response option best represented their mental state, just prior to the thought probe being presented. There were three response options: (1) “My thoughts were on task”, (2) “My thoughts were off task and I DO remember what I was thinking about”, and (3) “My thoughts were off task, but I DO NOT remember what I was thinking about”. Participants responded by pressing 1, 2, or 3 on the keyboard (for verbatim instructions, see Appendix A). A total of 24 thought probes were presented throughout the sustained attention to response task.

##### Questionnaires.

Following the experimental tasks, participants completed several questionnaires: The Spontaneous and Deliberate Mind-Wandering Scales (MWS/D; Carriere et al., 2013), The Mindful Attention Awareness Scale (MAAS; Brown & Ryan, 2003), and a demographics questionnaire. These were collected for exploratory purposes and analyzed in the Exploratory Analyses section.

#### Procedure.

Participants signed up for the experiment through the University of Manitoba’s SONA Psychology Research Participation Pool and completed the experiment online. Participants were first presented with the consent form, followed by an overview of the experimental tasks and instructions (see Appendix A). After the instructions, they completed the practice SART session, then the experimental session, and then the questionnaires. The order of these presented questionnaires was randomized for all participants.

### Results

As per the preregistration, we removed participants who had Go accuracy lower than 80% or No-Go accuracy lower than 20%. This removed a total of 14 participants. The data for the remaining participants are summarized in Table 1. While this metric was not employed as a criterion for participant removal—or preregistered as such—we also explored the incidence of participants missing five consecutive Go trials. The experiment was designed to pause if a participant missed five trials in succession. Among the remaining 139 participants, the majority experienced no pauses (*N* = 109), followed by those with one pause (*N* = 20), two pauses (*N* = 4), three pauses (*N* = 5), and four pauses (*N* = 1).

**Table 1.** A summary of the results from Experiment 1

Measure	High Demand					Low Demand				
	*M*	*SD*	Skew	Kurt	Alpha	*M*	*SD*	Skew	Kurt	Alpha
SART Performance										
Skill Index	1.25	0.42	−0.12	2.02		1.98	0.72	0.31	2.34	
ACC (GO)	0.97	0.03	−2.17	7.85		0.93	0.05	−0.23	2.04	
ACC (No-Go)	0.47	0.18	0.53	2.27		0.76	0.12	−0.76	3.64	
RT (Go)	376	63	1.02	3.75		428	143	1.44	5.21	
SART Probe Responses										
On Task	0.57	0.23	0.06	2.01		0.46	0.21	0.29	2.8	
Feeling	0.18	0.15	0.80	3.01		0.19	0.17	1.15	4.37	
Remembered	0.24	0.17	0.46	2.34		0.35	0.18	0.45	3.03	
Questionnaire Responses										
MWS	5.03	1.11	−0.90	4.65	0.73	4.92	1.32	−0.62	2.78	0.87
MWD	4.76	1.27	−0.55	2.56	0.81	4.57	1.31	−0.14	2.52	0.80
MAAS	3.56	0.79	0.25	3.58	0.88	3.61	0.78	−0.14	3.42	0.87

*Note*. *M* = Mean; *SD* = Standard Deviation; Skew = Skewness; Kurt = Kurtosis; Alpha = Cronbach’s Alpha; SART = Sustained attention to response task; MWS = Spontaneous Mind Wandering Scale; MWD = Deliberate Mind Wandering Scale; MAAS = Mindful Awareness and Attention Scale.

**Figure 1.**
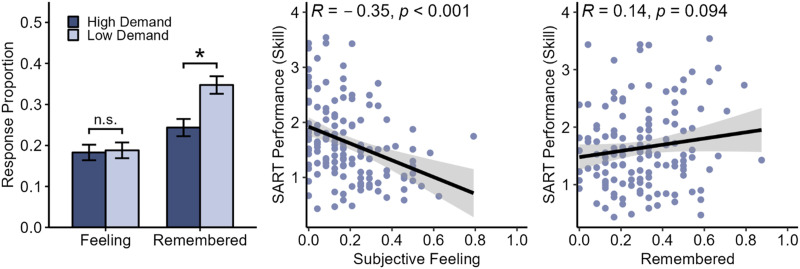
Results from Experiment 1. *Note*. Average subjective feeling of inattention (“Feeling” or “Subjective Feeling”) and remembered mind wandering (“Remembered) response proportions are plotted for the High and Low Demand groups (left). SART performance, in terms of “skill index” scores (accuracy on N-Go trials divided by average reaction time in Go trials), are plotted against response proportions of subjective feelings of inattention (center) and remembered (right) responses.

First, we examined whether our demand manipulation was successful by comparing performance across the low and high demand groups (see Figure 1). For each participant, we calculated a ‘skill index’ score (Jonker et al., 2013; Seli et al., 2016; Seli, Jonker, et al., 2013). The skill index combines commission errors and reaction times into a single efficiency score by dividing the accuracy on No-Go trials by the average reaction time on Go trials. The skill index score was developed to statistically control for speed-accuracy trade-offs—a strong negative relation between Go-trial reaction times and No-Go-trial accuracy—that are commonly observed in the SART (e.g., Dang et al., 2018; Head & Helton, 2013; Jonker et al., 2013; Seli et al., 2012). For instance, consider two participants who commit the same number of commission errors. If one of the participants had responded more quickly than the other, this faster participant would achieve a higher skill index score. This higher score signifies better overall performance, as it takes into account both speed and accuracy (see Seli, Jonker, et al., 2013).

Using a Welch’s *t*-test, we observed a significant difference between groups, with a higher skill index score for participants in the low demand group (*M* = 1.98) as compared to the high demand group (*M* = 1.25), Δ*M* = −0.72, 95% CI [−0.92, −0.52], *t*(137) = −7.08, *p* < .001, *d* = −1.2, 95% CI [−1.57, −0.84]. Next, we analyzed the overall frequency off-task thought by summing Remember and Subjective Feeling response proportions. Comparing the low and high demand groups using a Welch’s *t*-test, we find that the high demand group reported significantly less off-task thought (*M* = 0.43) than the low demand group (*M* = 0.54), Δ*M* = 0.11, 95% CI [0.03, 0.18], *t*(137) = 2.89, *p* = .005, *d* = 0.49, 95% CI [0.15, 0.83].

To determine how the demand manipulation influenced rates of Remember and Subjective Feeling responses, we analyzed response proportions using a mixed ANOVA with demand (high versus low) as a between-subjects factor and response (Remember versus Subjective Feeling) as a within-subjects factor. This analysis resulted in a significant main effect of demand, *F*(1, 137) = 8.33, *MSE* = 0.02, *p* = .005, ${\hat{\eta }}_{p}^{2}$ = .057, 90% CI [0.01, 0.13], a significant main effect of response, *F*(1, 137) = 26.08, *MSE* = 0.03, *p* < .001, ${\hat{\eta }}_{p}^{2}$ = .160, 90% CI [0.08, 0.25], and most importantly, a significant interaction between demand and response, *F*(1, 137) = 5.21, *MSE* = 0.03, *p* = .024, ${\hat{\eta }}_{p}^{2}$ = .037, 90% CI [0, 0.1]. Following up the significant interaction, we found that participants in the low demand group reported significantly more Remember mind wandering (*M* = 0.35) than participants in the high demand group (*M* = 0.24), Δ*M* = 0.10, 95% CI [0.04, 0.16], *t*(137) = 3.44, *p* < .001, *d* = 0.59, 95% CI [0.24, 0.93]. In contrast, there was no significant difference in the rate of Subjective Feeling responses between the low (*M* = 0.19) and high (*M* = 0.18) demand groups, Δ*M* = 0.01, 95% CI [−0.05, 0.06], *t*(137) = 0.20, *p* = .843, *d* = 0.03, 95% CI [−0.3, 0.37].

Finally, we were interested in whether SART performance was associated with Remember versus Subjective Feeling responses (see Figure 1). Correlating response rates with performance, we find a significant negative correlation between rates of Subjective Feeling responses and performance, *r* = −.35, 95% CI [−.49, −.19], *t*(137) = −4.33, *p* < .001, and no significant correlation between Remember mind wandering rates and performance, *r* = .14, 95% CI [−.02, .30], *t*(137) = 1.69, *p* = .094. Using Hittner et al.’s (2003; see also Zou, 2007) z-procedure for testing overlapping correlations based on dependent groups, we also find a significant difference between the two correlations, Δ*r* = 0.49, 95% CI [0.25, 0.71], *z* = 3.93, *p* < .05.

#### Exploratory Analyses.

Considering that the demand groups exhibited different patterns of response, which could lead to spurious correlations when combined, we re-analyzed the data by normalizing the skill index scores within each group. This approach controlled for group differences in scores while maintaining the full sample size. This analysis was not part of our pre-registered analysis plan. Correlating response rates with normalized performance, we find a significant negative correlation between rates of Subjective Feeling responses and performance, *r* = −.41, 95% CI [−.54, −.26], *t*(137) = −5.25, *p* < .001, and no significant correlation between Remember mind wandering rates and performance, *r* = −.04, 95% CI [−.21, .13], *t*(137) = −0.47, *p* = .640. Using the z-procedure for testing overlapping correlations based on dependent groups, we also find a significant difference between the two correlations, Δ*r* = 0.37, 95% CI [0.13, 0.59], *z* = 3.04, *p* < .05. Therefore, the pattern of results remained the same, after controlling for group differences.

Similarly, we also re-analyzed the data by filtering out thought probe responses with reaction times exceeding 30 seconds, aiming to exclude instances where participants were not actively engaged with the task and examine the robustness of our result. This removed 3.32% of thought probe responses. Although the full results can be found in the online supplementary materials, the pattern of significance remained the same across all analyses.

### Discussion

Experiment 1 investigated the novel distinction between remembered mind wandering and feelings of inattention. Our findings revealed that people frequently report subjective feelings of inattention, albeit less frequently than remembered experiences of mind wandering (20% vs. 25–35% of responses). Importantly, we identified two potentially important differences between subjective feelings of inattention and remembered experiences. First, we observed that our manipulation of task demands only affected rates of remembered experiences of mind wandering, with higher rates reported during low-demand tasks compared to high-demand tasks. In contrast, rates of subjective feelings of inattention did not differ between demand conditions. Second, we discovered that while subjective feelings of inattention were associated with poor performance, remembered experiences were not at all associated with performance. These results suggest that the subjective feelings of inattention and remembered mind wandering differ in meaningful ways and potentially reflect different underlying cognitive mechanisms.

For instance, changes in mind wandering that occur as a result of manipulating task demands is often taken as evidence for the attentional resource account, whereby excess resources are automatically allocated to mind wandering and decreases in task demands results in an increase in excess resources (Smallwood & Schooler, 2006). Here, we only find a change in remembered experiences, suggesting that these kinds of self-reports may reflect the allocation of excess resources to off-task thought. In contrast, the negative association between mind wandering and SART performance, a behavioural index of inattention, is often taken as evidence that mind wandering results from executive control failures (McVay & Kane, 2010). Interestingly, here, we found that only subjective experiences of inattention were associated with performance, which suggests that these self-reports may be related to failures in executive control.

In our view, these results are most consistent with Thomson et al.’s (2015) resource-control theory, which holds that mind wandering can be caused by either a failure of executive control *or* the allocation of excess resources. Our observed dissociations align with their predictions: Whereas, remembered mind wandering was sensitive to task-specific resource demands and unrelated to performance, subjective feelings of inattention were unaffected by task-specific resource demands, but were associated with poor performance. Interestingly, our results suggest that the phenomenology of these two kinds of mind wandering differ and can be measured simultaneously.

## EXPERIMENT 2

For Experiment 2, we completed a partial replication and extension of Experiment 1. In place of the sustained attention to response task, we adopted the metronome response task (Seli, Cheyne, et al., 2013). Some have argued that the sustained attention to response task is a poor behavioral measure of attention because of response strategies which can modulate speed-accuracy trade-offs (e.g., Mensen et al., 2022). The metronome response task overcomes such issues by having participants tap along to a metronome and quantifying performance as response variability rather than speed and accuracy. Like the sustained attention to response task, performance in the metronome response task has also been shown to be associated with mind wandering (e.g., Brosowsky et al., 2023; Seli, Cheyne, et al., 2013). During the metronome response task, we asked participants to report their mind wandering using the same thought probes as Experiment 1. In addition to the metronome task, we had participants complete an executive functioning task with confidence reports (Mazancieux et al., 2020). We included this task because it provided two additional individual differences measures. First, it provided a measure of executive functioning that was separate from our mind wandering task (see Head & Helton, 2018). Second, by including confidence reports, it provided a measure of metacognitive efficiency (M-Ratio), which indexes the extent to which participants can accurately monitor their own executive functioning performance. We speculated that the extent to which individuals can remember their mind wandering thoughts may be due to individual differences in general metacognitive ability.

### Methods

#### Sample Size Rationale.

As per our preregistration (https://osf.io/9kq63), we aimed to collect between 150 and 200 participants during a 3-week period, stopping either when we hit 200 participants or when the three-week data collection period ended—We successfully collected 200 participants before the deadline. We powered the experiment to detect differences in correlations, aligning with our analysis plan. We estimated that with 150 participants we would have approximately 80% power to detect differences between correlations of .2 and .3 and with 200 participants, 80% power to detect difference in correlations as small as .1 and .2.

#### Participants.

Two-hundred participants were recruited from the University of Manitoba SONA subject pool completed the experiment online for course credit. Participants were all students with ages ranging from 18 to 45 (mean = 20 [*SD* = 4.4], median = 19). When asked to indicate their gender using a free response, 113 identified as a woman or female, 79 as a man or male, 6 as non-binary (including “agender”, “non-binary”, “they/them” responses), and 2 participants did not provide a response. When asked about handedness, 161 indicated they were right-hand dominant, 22 were left-hand dominant, 10 indicated “both”, and 7 did not provide a response.

#### Task Materials.

All tasks were programmed in JavaScript using jsPsych (de Leeuw, 2015). All materials can be found online at https://osf.io/8rfbt/.

##### Metronome Response Task (MRT).

In the metronome response task, participants tap along to a steady aural beat using the space bar. Each trial was a total duration of 1300 ms: 650 ms of silence, a metronome tone lasting about 75 ms, and finally, 575 ms of silence. Participants completed a total of 560 trials. To elicit continuous engagement within the metronome response task, a warning symbol indicating consecutive missed trials was presented if participants missed five trials in a row and required participants to press the space bar to continue.

Within each block of 35 trials, a thought probe was randomly presented between trials 10 and 26. On each thought probe, participants were asked to indicate which response option best represented their mental state, just prior to the thought probe being presented. Thought probes were identical to those from Experiment 1. There were three response options: (1) “My thoughts were on task”, (2) “My thoughts were off task and I DO remember what I was thinking about”, and (3) “My thoughts were off task, but I DO NOT remember what I was thinking about”. Participants respond by pressing 1, 2, or 3 on the keyboard (for verbatim instructions, see Appendix A). A total of 13 thought probes were presented throughout the metronome response task.

##### Executive Functioning Task (Mazancieux et al., 2020).

On every trial, participants were presented with letter-number sequences of 5 symbols for 1000 ms. Half of the sequences had three letters and two numbers (e.g., “A1B2C”), while the other half had two letters and three numbers (e.g., “1A2B3”). After being presented with the string of letters and numbers, participants were presented with two response options and were instructed to choose an option that included the previously presented letters and the sum of the numbers. Distractor options were randomly generated by changing either 1 letter or the sum of all the numbers. For example, if a participant was presented with “A1B2C”, their response options would be “3ABC” or “3ADC”. In this case, the correct answer would be “3ABC” because it is the sum of all the numbers (“1 + 2 = 3”) and contains all the letters presented earlier (“A + B + C = ABC”).

Following each response, participants were asked to evaluate how confident they were in their selected answer. Along a percentage scale aligned horizontally, participants could report 0% (minimum confidence), 10%, 20%, 30%, 40%, 50%, 60%, 70%, 80%, 90%, or 100% (maximum confidence) by clicking on the corresponding label. During the instructional phase of the executive functioning task, it was explained to participants that a 0% confidence estimate indicated a guess. There was neither a time limit for participants to select a confidence judgment or a requirement to respond as quickly as possible.

The executive functioning task lasted about 8 minutes, with a total of 80 experimental trials and 80 confidence assessments. Prior to starting the experimental task, participants completed six practice trials, which included accuracy feedback, but did not include confidence judgments.

Task performance was quantified by calculating d-prime scores and metacognitive efficiency by calculating an M-Ratio score:

$$\text{MRatio}=\frac{\text{meta}\;d^{\prime }}{d^{\prime }}$$

Metacognitive efficiency is a measure of participants’ metacognitive sensitivity—an index of the accuracy of their confidence judgments—normalized by their actual performance. Thus, a score of 1 would indicate that their metacognitive sensitivity was as good as could be expected given their performance and less than 1 would indicate that their metacognitive sensitivity was worse than expected.

##### Questionnaires.

Following the experimental tasks, participants completed several questionnaires: The Spontaneous and Deliberate Mind-Wandering Scales (MWS/D; Carriere et al., 2013), The Mindful Attention Awareness Scale (MAAS; Brown & Ryan, 2003), and a demographics questionnaire. These were collected for exploratory purposes and analyzed in the Exploratory Analyses section.

#### Procedure.

Participants signed up for the experiment through the University of Manitoba’s SONA Psychology Research Participation Pool and completed the experiment online. Participants were first presented with the consent form, followed by an overview of the experimental tasks and instructions (see Appendix A). The order of the tasks was randomized, with roughly half the participants completing the metronome response task first, followed by the executive functioning task, and the other half receiving the reversed order. Following the behavioral tasks, participants completed the questionnaires in a randomized order.

### Results

As we did not pre-register participant exclusion criteria, we only excluded participants with missing data and extreme response patterns. This included three participants who were non-responsive during the MRT (missing > 90% of trials), two participants with M-Ratio scores 3 standard deviations above or below the mean, and two participants whose M-Ratio was incalculable with d-primes equal to 0—all together, we only removed a total of seven participants. The data for the remaining participants are summarized in Table 2.

**Table 2.** A summary of the results from Experiment 2

Measure	*M*	*SD*	Skew	Kurt.	Alpha
MRT Performance					
RRTv	8.57	0.75	0.52	3.44	
Omissions (%)	3.15	4.31	2.90	12.37	
MRT Probe Responses					
On Task	0.44	0.25	0.15	2.24	
Feeling	0.16	0.16	1.11	3.82	
Remembered	0.40	0.23	0.55	3.01	
EF Performance					
*d*-prime	2.03	0.92	0.18	3.15	
Meta-*d′*	2.14	1.26	0.63	3.49	
M-Ratio	1.05	0.53	−0.22	3.49	
Questionnaire Responses					
MWS	5.08	1.18	−0.46	2.69	0.79
MWD	4.81	1.23	−0.36	2.60	0.79
MAAS	3.43	0.79	0.20	2.99	0.88

*Note*. *M* = Mean; *SD* = Standard Deviation; Skew = Skewness; Kurt = Kurtosis; Alpha = Cronbach’s Alpha; RRTv = Log-transformed rhythmic response time variability; MRT = Metronome Response Time Task; EF = Executive Functioning Task; MWS = Spontaneous Mind Wandering Scale; MWD = Deliberate Mind Wandering Scale; MAAS = Mindful Awareness and Attention Scale.

#### MRT Performance.

**Figure 2.**
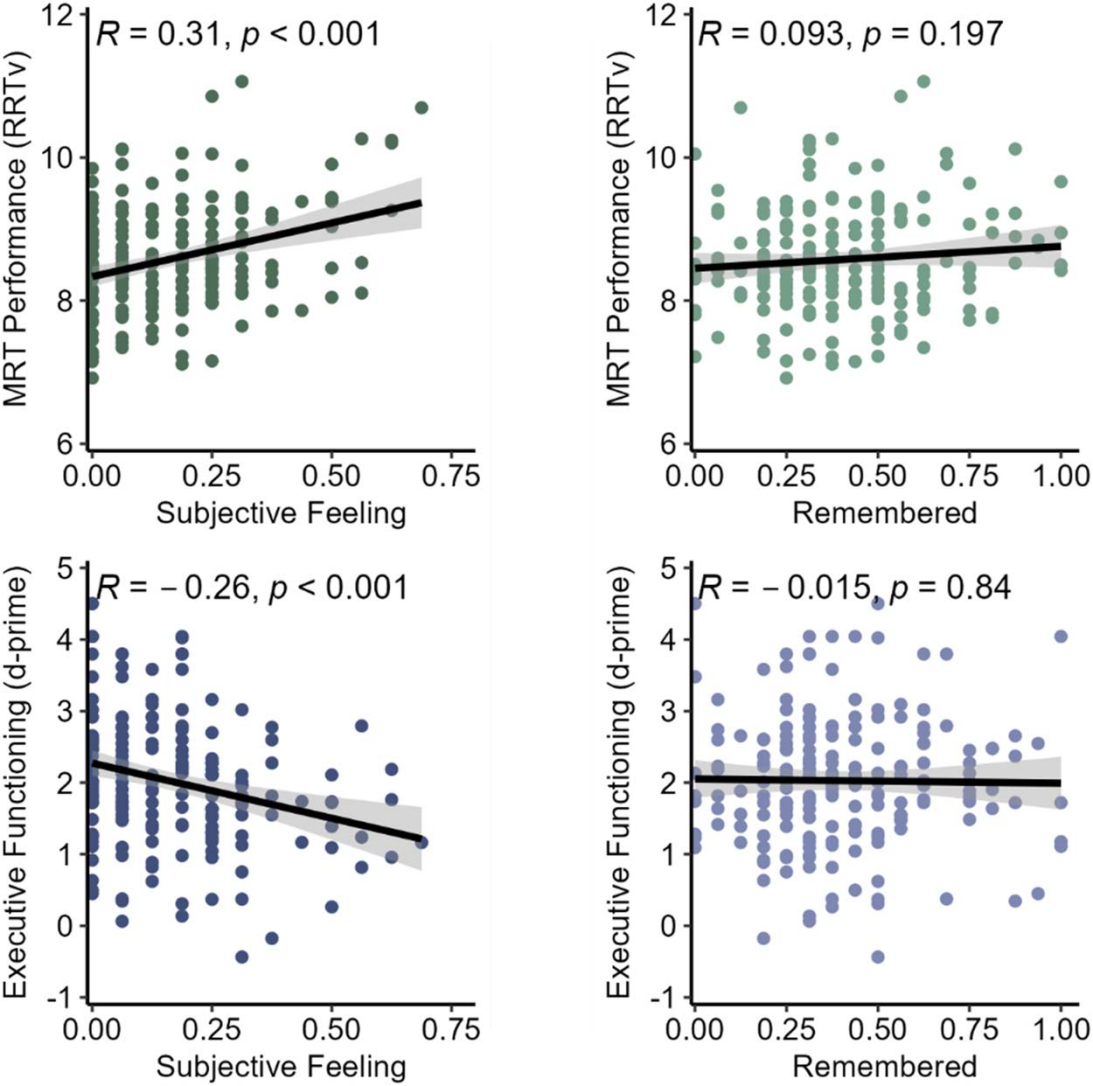
Results from Experiment 2. *Note*. Metronome response task (MRT) performance, in terms of rhythmic response time variance (RRTv), is plotted against response proportions for subjective feeling of inattention responses (top-left) and remembered mind wandering responses (top-right). Note that the higher RRTv is indicative of *worse* performance. On the bottom row, performance from the Executive Functioning task (d-prime scores) is plotted against response proportions for the subjective feeling of inattention responses (bottom-left) and remembered mind wandering responses (bottom-right); Here, lower scores are indicative of worse performance and worse executive functioning.

Performance on the MRT is indexed by rhythmic response time variability (RRTv). The RRTv is calculated as the average natural log of variance in response-time across 5-trial moving windows. First, we examined the associations between Remember and Subjective Feeling response rates and MRT performance (see Figure 2). Here, we found a significant correlation between RRTv and Subjective Feeling rates, *r* = .31, 95% CI [.18, .44], *t*(191) = 4.58, *p* < .001, no significant correlation with Remember mind wandering rates, *r* = .09, 95% CI [−.05, .23], *t*(191) = 1.30, *p* = .197, and a significant difference between the correlations, Δ*r* = −0.22, 95% CI [−0.43, −0.01], *z* = −2.05, *p* < .05.

#### Metacognitive Efficiency.

Next, we analyzed metacognitive ability, as measured by the M-Ratio scores and found no significant correlations between M-Ratio and Remember, *r* = −.01, 95% CI [−.15, .13], *t*(191) = −0.15, *p* = .880, or Subjective Feelings, *r* = −.10, 95% CI [−.24, .04], *t*(191) = −1.40, *p* = .164, mind wandering rates.

#### Executive Functioning.

Finally, we analyzed executive functioning (see Figure 2), as measured by the d-prime scores, and found a significant negative correlation with Subjective Feeling rates, *r* = −.26, 95% CI [−.39, −.13], *t*(191) = −3.78, *p* < .001, no significant correlation with Remember mind wandering rates, *r* = −.01, 95% CI [−.16, .13], *t*(191) = −0.20, *p* = .840, and a significant difference between correlations, Δ*r* = 0.25, 95% CI [0.03, 0.46], *z* = 2.27, *p* < .05.

#### Exploratory Analyses.

Although it was not part of our pre-registered analysis plan, we re-analyzed the data by filtering out thought probe responses with reaction times exceeding 30 seconds, aiming to exclude instances where participants were not actively engaged with the task to examine the robustness of our result. This removed 2.91% of thought probe responses. The full results can be found in the online supplementary material, however, the pattern of significance remained the same across all analyses, with the one exception that the difference in mind wandering-MRT performance correlations did not reach significance (Δ*r* = −0.19, 95% CI [−0.39, 0.02], *z* = −1.75, *p* = 0.08); Notably, the difference between mind wandering-executive functioning correlations remained significant (Δ*r* = 0.25, 95% CI [0.03, 0.46], *z* = 2.27, *p* < .05).

### Discussion

In Experiment 2, we successfully replicated the main dissociation observed in Experiment 1 using the metronome response task: poor performance was associated with subjective feelings of inattention and not remembered mind wandering. Moreover, this same dissociation was observed in our secondary executive functioning task, providing additional evidence that subjective feelings of inattention are driven by failures of executive control. Interestingly, individuals’ metacognitive efficiency (M-Ratio) did not predict rates of remembered mind wandering or subjective feelings of inattention which suggests that individual differences in remembering versus not remembering their thoughts are not the result of differences in their ability to monitor and track their cognitive states.

## EXPLORATORY ANALYSES

### Comparing Mind Wandering Reports Across Experimental Groups

**Figure 3.**
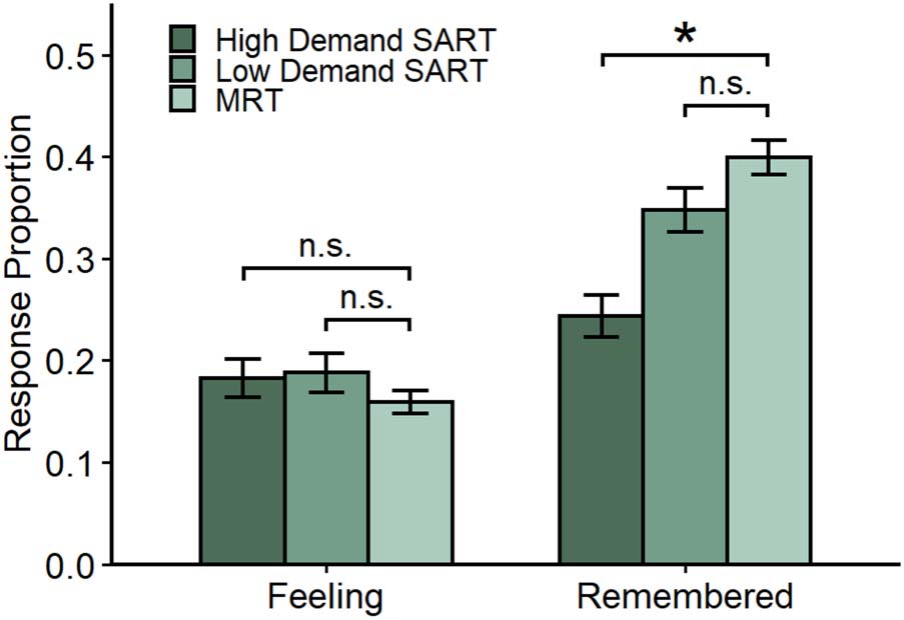
Thought probe responses across Experiments 1 and 2.

As an additional exploratory analysis, we compared mind wandering reports across Experiments 1 and 2 (see Figure 3). This analysis served as an additional test of the resource-dependency hypothesis, as the MRT should be comparable in terms of resource demands to the Low Demand SART and ought to differ from the High Demand SART. We submitted thought probe responses to a mixed ANOVA with group (High Demand SART, Low Demand SART, and MRT) as a between-subjects factor and response (Subjective Feelings versus Remember) as a within-subjects factor and found a significant interaction between group and response, *F*(2, 329) = 10.23, *MSE* = 0.04, *p* < .001, ${\hat{\eta }}_{p}^{2}$ = .059, 90% CI [0.02, 0.1]. We followed up the interaction by contrasting Remember and Subjective Feeling rates for the MRT group with the Low and High Demand SART groups. First, we found that the MRT group did not significantly differ in Subjective Feeling rates from the Low Demand, Δ*M* = 0.03, 95% CI [−0.01, 0.07], *t*(265) = 1.33, *p* = .185, *d* = 0.18, 95% CI [−0.09, 0.45], or High Demand, Δ*M* = 0.02, 95% CI [−0.02, 0.07], *t*(256) = 1.06, *p* = .292, *d* = 0.15, 95% CI [−0.13, 0.43], SART groups. Similarly, there was no significant difference in Remember mind wandering between the MRT group and the Low Demand SART group, Δ*M* = −0.05, 95% CI [−0.11, 0.01], *t*(265) = −1.74, *p* = .083, *d* = −0.24, 95% CI [−0.51, 0.03]. Critically, however, the MRT group did report significantly more Remember mind wandering than the High Demand SART group, Δ*M* = −0.16, 95% CI [−0.22, −0.09], *t*(256) = −5.02, *p* < .001, *d* = −0.72, 95% CI [−1.01, −0.43].

### Mind Wandering and Mindfulness

In both experiments, participants completed questionnaires assessing their mind wandering, as measured by the spontaneous and deliberate mind wandering scales (Carriere et al., 2013), and mindfulness, as measured by the mindful attention and awareness scale (Brown & Ryan, 2003). Although not the primary purpose of the current study, we also explored the associations between our novel state-level measures of mind wandering and trait mind wandering and mindfulness. Prior studies typically find low associations between state-level mind wandering reports and trait mind wandering and mindfulness self-reports (e.g., Kane et al., 2021). Therefore, for these exploratory analyses, we collapsed across experiments to increase our power to detect smaller associations (*N* = 332).

#### State- and Trait-Level Tendencies for Mind Wandering.

First, we assessed the associations between Remember and Subjective Feeling responses and trait-level mind wandering using partial correlations. We found that the frequency of Subjective Feeling responses was associated with spontaneous mind wandering while controlling for deliberate mind wandering, *r* = 0.16, 95% CI [0.05, 0.26], *t*(329) = 2.88, *p* < .05. However, the frequency of Subjective Feeling responses was not associated with deliberate mind wandering while controlling spontaneous mind wandering, *r* = −0.02, 95% CI [−0.12, 0.09], *t*(329) = −0.29, *p* = 0.77. Similarly, the frequency Remember mind wandering was associated with spontaneous mind wandering, while controlling for deliberate mind wandering, *r* = 0.11, 95% CI [0, 0.21], *t*(329) = 2, *p* < .05, and the frequency of Remember mind wandering, although marginal, was not associated with deliberate mind wandering, when controlling for spontaneous mind wandering, *r* = 0.1, 95% CI [0, 0.21], *t*(329) = 1.9, *p* = 0.06.

#### State-Level Mind Wandering and Trait-Level Tendencies for Mindfulness.

We also examined the associations between state-level mind wandering reports and trait mindfulness, as measured by the mindful attention and awareness scale. Here, we found that the frequency of Subjective Feeling responses was negatively associated with mindfulness, *r* = −.21, 95% CI [−.31, −.10], *t*(330) = −3.86, *p* < .001; and the frequency of Remember mind wandering was not significantly associated with mindfulness, *r* = −.10, 95% CI [−21, .01], *t*(330) = −1.83, *p* = .068.

## GENERAL DISCUSSION

In the current study we sought to better understand how people come to know whether they were mind wandering. We hypothesized two sources of information people might use to make such a judgment: Explicit memories of off-task thought and subjective feelings of inattention—a state in which individuals have a subjective experience that they were inattentive or mind wandering despite having no explicit recollection of the content of their thoughts. Across two experiments we observed that people often report having subjective feelings of inattention, albeit less frequently than remembered experiences of mind wandering (∼20% versus 25–40%).

We also observed theoretically meaningful dissociations across behavioural correlates of mind wandering. First, we found that whereas rates of remembered experiences of mind wandering varied with task demands, rates of subjective feelings did not (Exp. 1). Second, whereas subjective feelings of inattention were associated with poorer performance, remembered mind wandering rates were not (Exp. 1 and Exp. 2). Consistent with this last result, executive functioning, as measured by a secondary task, was associated with individuals’ rates of subjective feelings, but not remembered mind wandering (Exp. 2). Finally, our trait-level measures of mind wandering and mindfulness did not dissociate between our state-level measures, which suggests that the distinction between remembered mind wandering and feelings of inattention is not redundant with the distinction between spontaneous and deliberate mind wandering. However, replicating prior research (e.g., Kane et al., 2021) we found that spontaneous mind wandering was associated with state-level mind wandering, though, we observed larger associations with subjective feelings of inattention than remembered mind wandering. Interestingly, mindfulness was only associated with subjective feelings of inattention, and not remembered mind wandering.

Taken together, the results of our study suggest that people do indeed rely on different sources of information to determine whether they were mind wandering, sometimes relying on explicit memories and sometimes on subjective feelings. These findings are consistent with a larger body of research on metacognition, which has identified similar dissociations in judgments about memory (e.g., Whittlesea & Williams, 2000, 2001a, 2001b) and learning (Koriat, 2007)—sources of inspiration for the current framework.

Our study also contributes to the current theoretical explanations of mind wandering, providing support for Thomson et al.’s (2015) Resource-Control Account. This account suggests that mind wandering can arise from either an excess of attentional resources or a failure of executive control, which are not mutually exclusive and have differing behavioral correlates. The dissociations we observed in our study align with this account. Specifically, as predicted by the Resource-Control Account, remembered experiences were resource-dependent and did not impair performance, suggesting they may result from an excess of resources. In contrast, rates of subjective feelings of inattention were independent of attentional resources but associated with poor performance and executive functioning, suggesting they may stem from failures of executive control. Critically, our novel thought probe seems to be able to dissociate these two causes of mind wandering, potentially enabling researchers to measure their influence simultaneously.

From a methodological perspective, these results have important implications for measuring mind wandering, and researchers should consider how a participant would—or should—respond to their probes when they have a feeling of inattention without an explicit memory. Thought probes are typically forced-choice, requiring participants to categorize their thoughts based on the content—which, of course, assumes that participants *can* remember the content. In many cases, it is unclear how a participant should categorize a ‘feeling of inattention’, and depending on the instructions given, even a simple on versus off task categorization may create ambiguity and systematic bias across studies. For instance, one might assume that a feeling of inattention should be sufficient for selecting “off task.” However, how the instructions are phrased might influence whether these experiences are categorized as off task. Arabacı and Parris (2018), for example, specifically instructed participants to only choose off task when they explicitly remember what their thoughts were and in cases when they have no memory, to select “on task”. More recently, in an impressive effort, Kane et al. (2021) examined the reliability of a thought probe that asks participants to categorize their thoughts’ topic given a predefined set of topics. However, this measure also does not clarify what participants should do when they cannot remember their thoughtbut believe they were inattentive. Given the dissociations we observed, such discrepancies across studies may help explain failures to replicate the common associations between behavior and self-reported mind wandering, or in the very least, introduce additional measurement noise within and across studies.

Finally, it is worth considering how our novel framework compares to previous conceptualizations of mind wandering and their associated thought probes. Perhaps the most relevant to our ‘feelings of inattention’ is the notion of ‘mind blanking’, where participants are asked to indicate whether their ‘mind was blank’ or had a ‘gap in consciousness’ just prior to a thought probe (e.g., Ward & Wegner, 2013). There is some similarity of course, since if one’s mind was ‘blank’ then they likely will have no memory of their inattention episode. However, the lack of memory does not necessitate a blank mind. A person, for example, might report being off-task and unable to recall their thoughts simply because they have forgotten their thoughts, not because they were rendered unconscious for the episode or had “no thoughts”^3^. Similarly, many measures of mind blanking do not ask participants to indicate whether they believed they were off task, or inattentive, when their mind was blank. It may be possible that someone lacks conscious awareness during their experience, but still believes they were on task or were performing at a high level. This possibility, to our knowledge, has not been explored, but bears resemblance to ideas about ‘flow states’ (Csikszentmihalyi, 1988; see also, Brosowsky et al., 2021b) and effortless concentration (Marty-Dugas & Smilek, 2019). Empirically, there is also reason to believe these two measures reflect different mechanisms. Whereas here we find that subjective feelings of inattention reliably predict task performance, previous studies investigating mind-blanking have typically found that off-task thought predicted performance and rates of mind-blanking *did not* (reading tasks; Ward & Wegner, 2013, SART; Beikmohamadi & Meier, 2022; Stawarczyk & D’Argembeau, 2016). However, it is also important to note that the current study’s objective was not to empirically distinguish feelings of inattention from mind blanking. While we argue that they are conceptually separate, this is a limitation of the study, and empirically differentiating these two aspects would enhance our understanding of both. Additionally, it may be significant, both theoretically and empirically, to further differentiate in our measurement between ‘feelings of inattention’ that stem from mind blanking and those that do not.

Our theoretical approach also differs in that we begin with a general theory of how metacognitive judgments are made and attempt to map behavior within our theoretical framework. Importantly, we have not made assumptions about how a ‘feeling of inattention’ might be brought about. In fact, we would argue there are many reasons why a person might feel inattentive, despite not being able to recall their thoughts. For instance, if one’s performance were suddenly disfluent without an apparent cause, one might come to have a feeling of inattention (e.g., Brosowsky et al., 2021a). A fruitful path forward would involve further elucidating the causes of feelings of inattention to better explain why individuals believe they were inattentive, even when they cannot recall the contents of their thoughts.

Similarly, “feelings of inattention” may be related to confidence in mind wandering judgments. Some previous studies have involved participants making a mind wandering judgment, followed by a rating of their confidence in that judgment. For example, Seli et al. (2015) found that mind wandering judgments made with high confidence showed a stronger relationship with performance than those made with low confidence. However, subsequent studies failed to find any association between confidence and performance (Anderson et al., 2021; Meier, 2018). Kane et al. (2021) observed that participants were more confident in judgments related to the content of mind wandering than in those pertaining to ‘intentionality’ or ‘depth’. Furthermore, they observed that confidence varied in relation to the depth of mind wandering judgments, with deeper mind wandering typically associated with lower confidence. Within this framework, feelings of inattention might reflect mind wandering experiences typified by ‘low confidence’, whereas remembered off-task thoughts could align with ‘high confidence’ mind wandering. Intriguingly, the presence or absence of memory might play a pivotal role in shaping the confidence levels associated with mind wandering judgments. Exploring the connection between mind wandering confidence and feelings of inattention could provide valuable insights in future studies.

There are several notable limitations in the current study. First, both experiments were conducted online, subjecting our methods to the constraints typical of online data collection. Specifically, we could not monitor participants’ full engagement with the experiment or whether they took breaks. In retrospect, we should have designed the task to impose a time limit on probe responses and a pause in the task following a probe response to discourage participants from taking a break at the probe screen. Additionally, we did not pre-register the criteria for participant removal in Experiment 2. While most results were robust across various data analysis decisions, this was not universally the case (as detailed in the online supplementary analyses). Therefore, although the results of the two experiments align with each other, further replications would bolster the claims made here, and the results should be regarded as preliminary evidence highlighting the importance of measuring feelings of inattention.

A second limitation of this study involves the time-on-task and the number of probes presented. Both tasks were relatively brief, lasting 15–25 minutes, and the number of probes within each task may be considered low, especially since they were divided among three response options (E1 = 24 probes, E2 = 16 probes). Regarding the quantity of probes, a recent study (Welhaf et al., 2023) concluded that as few as 8 probes could reliably measure off-task thought. However, in our study, we measured two types of mind wandering instead of one, which limits the applicability of their findings. Time-on-task is also a crucial determinant of inattention and mind wandering, and the outcomes might vary when using longer tasks. Furthermore, future research should explore how feelings of inattention, as opposed to remembered off-task thought, evolve with increased time-on-task. We observed dissociations in this study, suggesting that these forms of mind wandering might differ in their temporal changes, which could further illuminate the theoretical mechanisms underlying each type of inattention experience (for example, see Thomson et al., 2015).

## CONCLUDING REMARKS

Across two experiments, we have established that individuals often have experiences where they subjectively evaluate their attentional state as “off task” or experiencing mind wandering yet are unable to recall the specific content of their thoughts. From a methodological perspective, the observed dissociations underscore the importance of differentiating between remembered experiences and subjective feelings of inattention during mind wandering assessments. Future research should also place emphasis on providing clear instructions to participants on how to respond in instances where they cannot explicitly recall their thoughts but still perceive themselves to be off task. From a theoretical perspective, our results offer novel evidence that individuals rely on more than just the content of their thoughts when making metacognitive judgments about their mind wandering. This study extends an extensive body of research on metacognition (e.g., Koriat, 2007; Whittlesea et al., 1990; Whittlesea & Williams, 2001a) into the mind wandering domain and lays the groundwork for further investigations into the metacognitive mechanisms that underlie judgments about mind wandering. Finally, the results of the current study suggest that differentiating between remembered mind wandering and subjective feelings of inattention could significantly contribute to the refinement of our theories on mind wandering.

## ACKNOWLEDGMENTS

This research was supported by a Discovery Grant RGPIN-2022-04498 from the Natural Sciences and Engineering Research Council of Canada awarded to Nicholaus P. Brosowsky.

## FUNDING INFORMATION

This research was supported by a Discovery grant from the Natural Sciences and Engineering Research Council of Canada (NSERC) awarded to N. P. Brosowsky.

## AUTHOR CONTRIBUTIONS

N.M.: Conceptualization; Investigation; Methodology; Writing – original draft; Writing – review & editing. U.B.F.: Conceptualization; Investigation; Methodology; Writing – review & editing. N.B.: Conceptualization; Formal analysis; Methodology; Supervision; Writing – original draft; Writing – review & editing.

## DATA AVAILABILITY STATEMENT

All data, materials, and code are available at https://osf.io/8rfbt/.

## Notes

^1^ Historically, many studies have asked participants to report their experiences over extended periods of time, such as reporting whether or how much they mind wandered during previous 2 to 15 minutes (e.g., Antrobus et al., 1966; Filler & Giambra, 1973; Giambra, 1989). Modern research has largely moved away from this method because it is assumed that such reports are less reliable due to forgetting and mental-aggregation biases (see Kane et al., 2021)—though, we note the lack of research regarding this issue.^2^ There was an error in our pre-registered power analysis, where Cohen’s *f* of .1 should have been reported as .15. Regardless, we collected a sufficiently larger sample size to detect medium-sized interaction effects following our pre-registered sample size of 75 participants per group.^3^ Ward and Wegner (2013; pg. 5) instructed participants that “The only difference between mind-wandering and mind-blanking is that in mind-wandering you’re thinking about something else, and in mind-blanking you aren’t”.

## APPENDIX A: VERBATIM THOUGHT PROBE INSTRUCTIONS FROM EXPERIMENT 1 AND EXPERIMENT 2

“Throughout the task, we will also periodically ask you about the content of your thoughts while you were performing the task. Every once in a while, the task will temporarily stop, and you will be presented with a screen asking you to indicate whether you were “ON TASK” or “OFF TASK”

Being ON TASK means that, just before the screen appeared, you were focused on completing the task and were not thinking about anything unrelated to the task. Some examples of ON TASK thoughts include thoughts about your performance on the task, thoughts about the digits, or thoughts about your response.

On the other hand, being OFF TASK means that, just before the screen appeared, you were thinking about something completely unrelated to the task. Some examples of OFF TASK thoughts include thoughts about what to eat for dinner, thoughts about plans you have with friends, or thoughts about an upcoming test. It is perfectly normal to think about things that are not related to the task.

We would also like to know how often you remember what your OFF-TASK thoughts were about. Sometimes when you find yourself thinking about something unrelated to the task you can recall exactly what it was you were thinking about.

Other times, however, you may not remember exactly what you were thinking about, but still you **know** that you weren’t focused on the task. Again, this is perfectly normal. To answer these questions, periodically, we will pause the experiment and present the following screen. You will use the keyboard to respond.

“Which of the following responses best characterizes your mental state just prior to the presentation of this screen?

1. [PRESS 1] ON TASK
2. [PRESS 2] OFF TASK and I remember what I was thinking about.
3. [PRESS 3] OFF TASK but I do **NOT** remember what I was thinking about.”

Please try your best to honestly assess your thoughts and choose a response that best describes your thoughts at the time when we ask. We will briefly review the response options again on the following pages.

“[PRESS 1] ON TASK” You should choose this response option when your thoughts just before the screen appeared were related to the task (e.g., thoughts about your performance, thoughts about the digits, or thoughts about your response). In other words, you were focused on the task.

“[PRESS 2] OFF TASK and I remember what I was thinking about.” You should choose this option when, just before the screen appeared, you can recall the content of your thoughts, and they were unrelated to the task (e.g., thoughts about what to eat for dinner, thoughts about plans with friends, or thoughts about an upcoming test). In other words, you find yourself thinking about something other than the task and can recall the details of those thoughts.

“[PRESS 3] OFF TASK but I do **NOT** remember what I was thinking about.” You should choose this option if you cannot recall the details of your thoughts just before the screen appeared, but you know that your thoughts were not on the task. In other words, you should choose this option if you know you were NOT focused on the task, but you cannot recall the details or content of your thoughts.”
